# Inverse‐Perovskite Ba_3_ *B*O (*B* = Si and Ge) as a High Performance Environmentally Benign Thermoelectric Material with Low Lattice Thermal Conductivity

**DOI:** 10.1002/advs.202307058

**Published:** 2023-12-25

**Authors:** Xinyi He, Shigeru Kimura, Takayoshi Katase, Terumasa Tadano, Satoru Matsuishi, Makoto Minohara, Hidenori Hiramatsu, Hiroshi Kumigashira, Hideo Hosono, Toshio Kamiya

**Affiliations:** ^1^ MDX Research Center for Element Strategy International Research Frontiers Initiative Tokyo Institute of Technology 4259 Nagatsuta, Midori Yokohama 226‐8501 Japan; ^2^ Research Center for Magnetic and Spintronic Materials National Institute for Materials Science 1‐2‐1 Sengen Tsukuba Ibaraki 305‐0047 Japan; ^3^ Research Center for Materials Nanoarchitectonics National Institute for Materials Science 1‐1 Namiki Tsukuba, Ibaraki 305‐0044 Japan; ^4^ Research Institute for Advanced Electronics and Photonics National Institute of Advanced Industrial Science and Technology Tsukuba Ibaraki 305‐8568 Japan; ^5^ Laboratory for Materials and Structures Institute of Innovative Research, Tokyo Institute of Technology 4259 Nagatsuta Midori, Yokohama 226‐8501 Japan; ^6^ Institute of Multidisciplinary Research for Advanced Materials Tohoku University Sendai 980‐8577 Japan

**Keywords:** electronic transport, material design, phonon scattering, semiconductor, thermoelectric material

## Abstract

High energy‐conversion efficiency (*ZT*) of thermoelectric materials has been achieved in heavy metal chalcogenides, but the use of toxic Pb or Te is an obstacle for wide applications of thermoelectricity. Here, high *ZT* is demonstrated in toxic‐element free Ba_3_ *B*O (*B* = Si and Ge) with inverse‐perovskite structure. The negatively charged *B* ion contributes to hole transport with long carrier life time, and their highly dispersive bands with multiple valley degeneracy realize both high p‐type electronic conductivity and high Seebeck coefficient, resulting in high power factor (PF). In addition, extremely low lattice thermal conductivities (*κ*_lat_) 1.0–0.4 W m^−1^ K^−1^ at *T* = 300–600 K are observed in Ba_3_ *B*O. Highly distorted O–Ba_6_ octahedral framework with weak ionic bonds between Ba with large mass and O provides low phonon velocities and strong phonon scattering in Ba_3_ *B*O. As a consequence of high PF and low *κ*_lat_, Ba_3_SiO (Ba_3_GeO) exhibits rather high *ZT* = 0.16–0.84 (0.35–0.65) at *T* = 300–623 K (300–523 K). Finally, based on first‐principles carrier and phonon transport calculations, maximum *ZT* is predicted to be 2.14 for Ba_3_SiO and 1.21 for Ba_3_GeO at *T* = 600 K by optimizing hole concentration. Present results propose that inverse‐perovskites would be a new platform of environmentally‐benign high‐*ZT* thermoelectric materials.

## Graphical abstract

High thermoelectric performance is demonstrated in inverse‐perovskite Ba_3_ *B*O (*B* = Si and Ge). The Ba_3_ *B*O shows high power factor due to highly dispersive valence bands with multiple valley degeneracy, and also exhibits low lattice thermal conductivity due to the soft framework of O−Ba_6_ octahedra with ionic O−Ba bonds. Inverse‐perovskite oxides would be a potential high‐*ZT* thermoelectric material without toxic elements.

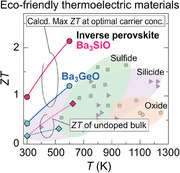

## 1 Introduction

Due to the recently increasing energy crisis, there has been increasing attention to thermoelectric technology for power generation using waste heat energy.^[^ ^1^, ^2^, ^3^ ^]^ The efficiency of thermoelectric energy conversion is governed by the dimensionless figure of merit (*ZT*), defined as *ZT* = *S* ^2^·*σ*·*T*·*κ* ^–1^, where *T* is the absolute temperature, *S* is the Seebeck coefficient, *σ* is the electronic conductivity, and *κ* is the thermal conductivity of the thermoelectric materials.^[^ ^4^, ^5^, ^6^ ^]^ The product *S* ^2^ *σ* is known as the power factor (PF), and the *κ* includes the contributions from electronic (*κ*_ele_) and lattice (*κ*_lat_) heat conduction. Therefore, high *ZT* thermoelectric materials should exhibit large *S* and high *σ* to obtain high PF, as well as low *κ* to create a large temperature gradient. So far, the high *ZT* has been demonstrated mainly in heavy metal chalcogenides, such as Bi_2_Te_3_, PbTe, and GeTe,^[^ ^7^, ^8^, ^9^ ^]^ which possess low *κ*_lat_, but the use of toxic elements, such as Pb and Te, is not preferred for wide applications of thermoelectricity. There are many efforts on the development of environmentally benign thermoelectric materials, such as oxides, silicides, and sulfides,^[^ ^10^, ^11^, ^12^, ^13^, ^14^ ^]^ but further exploration of novel material systems is demanded for improving the thermoelectric performance.

**Figure 1:**
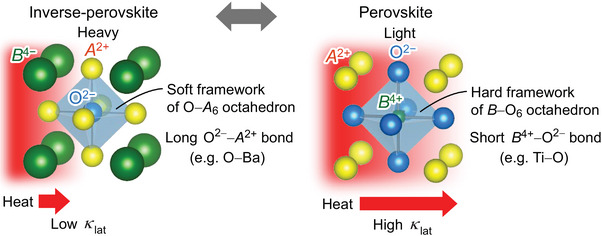
Schematic illustration of crystal structures and phonon transport in inverse‐perovskite *A*_3_ *B*O (left) and normal perovskite *AB*O_3_ (right). The normal perovskite structure of *AB*O_3_ (e.g., SrTiO_3_) is built with the hard framework of *B*−O_6_ octahedron with short *B*−O bonds, providing high‐density packing structure of the light element O^2−^ ions. In contrast, the inverse‐perovskite structure of *A*_3_ *B*O (e.g., Ba_3_ *B*O (*B* = Si and Ge)) is constructed from the soft framework of O−*A*_6_ octahedron with long O−*A* bonds, providing the high‐density packing structure of heavy *A* ^2+^ ions. The lattice thermal conductivity (*κ*_lat_) of normal perovskite *AB*O_3_ is usually high, while the largely contrasting structure characteristics are expected to lead the large reduction of *κ*_lat_ in inverse‐perovskite *A*_3_ *B*O.

We herein focus on inverse‐perovskite oxides as potential environmentally benign thermoelectric materials without toxic elements. The inverse‐perovskite oxides are represented by chemical formula of *A*_3_ *B*O with formal ion charges of *A* ^2+^ (alkaline earth = Ca^2+^, Sr^2+^, Ba^2+^), *B* ^4–^ (the p‐block 14 group = Si^4−^, Ge^4−^, Sn^4−^, Pb^4−^), and O^2−^.^[^ ^15^, ^16^, ^17^, ^18^, ^19^, ^20^ ^]^ It crystallizes in the inverse‐perovskite structure that has an inverted cation and anion sites in comparison to the normal perovskite oxide *AB*O_3_ (*A* ^2+^ *B* ^4+^O^2−^) such as SrTiO_3_ (**Figure** **1**). In the normal perovskite structure, *B* ^4+^ cation occupies the body‐centered site of the pseudo‐cubic unit cell and O^2−^ anion occupies the face‐centered sites, forming a *B*–O_6_ octahedron. *A* ^2+^ cation occupies the vertex sites of the unit cell. On the other hand, in the inverse‐perovskite structure, O^2−^ anion occupies the body‐centered site and the *A* ^2+^ cation occupies the face‐centered sites, forming an O−*A*_6_ octahedron. *B* ^4−^ anion occupies the vertex sites. *ZT* of perovskite SrTiO_3_ is usually limited to ∼0.1 due to its high *κ*_lat_ ≈10 W m^−1^ K^−1^ at room temperature (RT),^[^ ^21^ ^]^ originating from the hard framework of the Ti–O_6_ octahedra with the strong Ti–O bonds. In contrast, the inverse‐perovskite structure is constructed from the soft framework of the O–*A*_6_ octahedra because of the larger *A* ^2+^ ion than *B* ^4+^ ion and the consequent longer O–*A* bonds. From another point of view, normal perovskite SrTiO_3_ is formed by the high‐density packing structure of the light element O^2−^ ions, while inverse‐perovskite *A*_3_ *B*O is formed by the high‐density packing structure of heavier *A* ^2+^ ions. These largely contrasting structural characteristics let us expect a large reduction of *κ*_lat_ in inverse‐perovskite *A*_3_ *B*O. The most distinctive feature of inverse‐perovskite *A*_3_ *B*O is that the *B* ion is negatively charged, which actively contributes to hole conduction; the localized O 2*p* state forms valence band maximum (VBM) in conventional oxides including normal perovskite oxides, while spatially spread *p* orbital of large‐size *B* ^4−^ anion (ion radius: > 2 Å of *B* ^4−^, 1.4 Å of O^2−^) forms VBM in inverse‐perovskite oxide,^[^ ^22^ ^]^ which can realize high hole mobility and *σ*. Our work is hence motivated by the expectation that the inverse‐perovskite oxide would be a potential candidate for high *ZT* thermoelectric materials.

The *A*_3_ *B*O with *B* = Sn and Pb adopts a high‐symmetry cubic structure (*Pm*‐3*m*) and exhibits unique Dirac electronic structures with high carrier mobility, being expected as a new class of topological crystalline insulators and superconductors.^[^ ^22^, ^23^, ^24^, ^25^, ^26^ ^]^ However, their narrow bandgaps (theoretical bandgaps < 0.2 eV) limit their thermoelectric properties because the compensation by the coexistence of electrons and holes leads to low *S*, resulting in low PF at high temperatures.^[^ ^27^ ^]^ On the other hand, theoretical studies proposed that bandgap is sensitive to structural distortion, which enhances the thermoelectric properties of inverse‐perovskite *A*_3_ *B*O.^[^ ^28^, ^29^, ^30^ ^]^ By replacing the *B* site with smaller Si and Ge ions, the cubic structure is distorted to an orthorhombic lattice in agreement with a smaller tolerance factor ($t=\frac{r_{B}+r_{A}}{2^{1/2}\left(r_{O}+r_{A}\right)}$) of an inverse‐perovskite structure, where *r_A_*, *r_B_*, *r*_O_ are ionic radii for *A*, *B*, O ions.^[^ ^28^, ^31^ ^]^ The orthorhombic Ca_3_SiO (Ca_3_GeO) with *t* = 0.937 (0.948) takes the space group of *Imma* and increases the bandgap to 0.7–0.9 eV.^[^ ^28^, ^32^ ^]^ The thermoelectric properties were experimentally measured for Ca_3_SiO and Ca_3_GeO bulk polycrystals, which show low *κ*_lat_ = 1.0–1.9 W m^−1^K^−1^ at RT but the obtained *ZT* are limited to less than 10^−5^ because the properties were measured with cold‐pressed bulks with large amount of CaO impurity (24%–32% in the Ca_3_SiO sample and 8%–10% in the Ca_3_GeO sample).^[^ ^33^ ^]^

**Figure 2:**
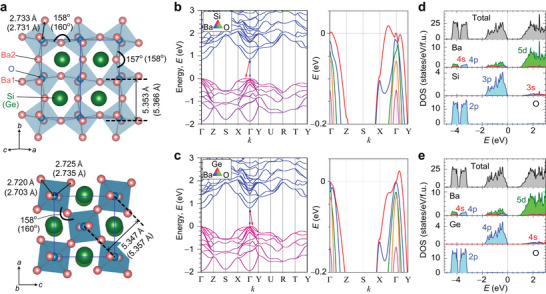
Crystal structure and electronic structure analyses of inverse‐perovskite Ba_3_ *B*O (*B* = Si and Ge). a) Crystal structures of Ba_3_SiO (Ba_3_GeO) with space group of *Pnma*. The top and the bottom panels are the side view of (101) plane and the top view of (010) plane, respectively. b,c) Electronic band structures and d,e) partial density of states for b,d) Ba_3_SiO and c,e) Ba_3_GeO. The right panels of (b,c) are enlarged views of valence band maximum.

Here, we synthesized high‐purity bulk polycrystals of highly distorted Ba_3_ *B*O (*B* = Si and Ge) with *t* = 0.908 and 0.918, which crystalize in orthorhombic inverse‐perovskite structures (space group: *Pnma*) with the pronounced tilting and twisting of the O−Ba_6_ octahedra (**Figure** **2**a). The theoretical bandgaps are 0.86 eV for Ba_3_SiO and 0.80 eV for Ba_3_GeO. The undoped samples showed p‐type degenerate conduction with hole concentrations ≈4.8 × 10^18^ cm^−3^ for Ba_3_SiO and ≈6.2 × 10^19^ cm^−3^ for Ba_3_GeO at RT. The bulk samples exhibited low *κ*_lat_ of 1.00 W m^−1^ K^−1^ for Ba_3_SiO and 0.77 W m^−1^ K^−1^ for Ba_3_GeO, which are lower than 1.7–2.0 W m^−1^ K^−1^ of Bi_2_Te_3_ and PbTe bulks at RT. Ba_3_SiO and Ba_3_GeO bulks exhibited relatively high *ZT* = 0.16 and 0.35 at RT, respectively, and the *ZT* value increased up to 0.84 for Ba_3_SiO bulk at *T* = 623 K and 0.65 for Ba_3_GeO bulk at *T* = 523 K. We systematically investigated the electronic and phonon transport properties of Ba_3_ *B*O by using first‐principles calculations to clarify the underlying physical mechanisms for their low *κ*_lat_ and the potential of thermoelectric *ZT*.

## 2 Results and Discussion

### 2.1 Crystal Structure and Electronic Structure Analyses

The bulk polycrystals of Ba_3_ *B*O (*B* = Si and Ge) were synthesized by high‐temperature solid‐state reactions of 2Ba + Si(Ge) +BaO → Ba_3_SiO(Ba_3_GeO). From X‐ray diffraction (XRD) measurements, a small amount of BaO impurity (7.3 mol%) is detected for the Ba_3_GeO bulk, and the weak unidentified diffraction peaks are observed for the Ba_3_SiO bulk (Figure S1, Supporting Information, and CCDC 2291770 and 2291771 are the supplementary crystallographic data for this paper). Microstructure analysis by a field‐emission scanning electron microscopy (FE‐SEM) shows that the bulks are composed of sintered grains with an average grain size of ≈10 µm with some pores (Figure S2, Supporting Information), resulting in the sintered density of 80–87%. Energy dispersive X‐ray spectroscopy (EDS) mapping confirms the uniformity of the chemical composition of Ba, Si (Ge), and O over the grain region. The orthorhombic lattice parameters estimated by Rietveld analysis of XRD patterns are *a* = 7.581 Å, *b* = 10.706 Å, *c* = 7.543 Å for Ba_3_SiO and *a* = 7.592 Å, *b* = 10.732 Å, *c* = 7.559 Å for Ba_3_GeO. Lattice volume is expanded from 612.261 Å^3^ of Ba_3_SiO to 615.896 Å^3^ of Ba_3_GeO because of the slightly larger ion radius of 2.08 Å for Ge^4−^ anion than 2.04 Å of Si^4−^ anion.^[^ ^31^ ^]^ The pseudo‐cubic lattice parameters of the orthorhombic unit cell for Ba_3_SiO (Ba_3_GeO) are *b*/2 = 5.353 Å (5.366 Å) and $\sqrt{a^{2}+c^{2}}$ = 5.347 Å (5.357 Å), indicating the O−Ba_6_ octahedra are slightly elongated along the *b*‐axis (Figure 2a). The orthorhombic distortion splits the Ba sites to the non‐equivalent Ba1 and Ba2 sites. The O−Ba_6_ octahedra are tilted and twisted around all three octahedral axes, where the apical O−Ba2−O and basal O−Ba1−O bond angles for Ba_3_SiO (Ba_3_GeO) are 157^o^ (158^o^) and 158^o^ (160^o^), showing distinct deviations from 180^o^ of the cubic lattice.

Figure 2b‐e summarizes electronic band structures and density of states (DOSs) of Ba_3_ *B*O calculated by the VASP^[^ ^34^, ^35^ ^]^ code with Heyd–Scuseria–Ernzerhof (HSE) hybrid functional.^[^ ^36^ ^]^ The conduction band minimum (CBM) and VBM located around the Γ point (left panels of Figure 2b,c), where the difference in the *k* vector between direct and indirect bandgap (*E*_g_) is small, as indicated by the blue and the red arrows. The *E*_g_ are calculated to be 0.86 eV for Ba_3_SiO and 0.80 eV for Ba_3_GeO, which are a little larger than the experimentally measured *E*_g_ of 0.62 eV and 0.58 eV (See diffuse reflectance spectra in Figure S3, Supporting Information), due to the *E*_g_ overestimation by HSE hybrid functional. The atomic charges estimated by the Bader charge analysis are Ba^+1.15^_3_Si^–2.02^O^−1.45^ and Ba^+1.14^_3_Ge^–1.99^O^−1.45^, confirming the anionic states of Si and Ge ions. The CBM is mainly contributed by the Ba 5*d* state with only one single valley at the Γ point, while the VBM arises primarily from the Si 3*p* (Ge 4*p*) state (Figure 2d,e). Specifically, the valence bands around the Γ point are composed of one flat band and four highly dispersive bands nearly degenerating within the 0.15 eV energy range below the Fermi level (right panels of Figure 2b,c). On the other hand, O 2*p* state located at a deeper energy level contributing little to carrier transport.

### 2.2 Carrier Transport Properties

**Figure 3:**
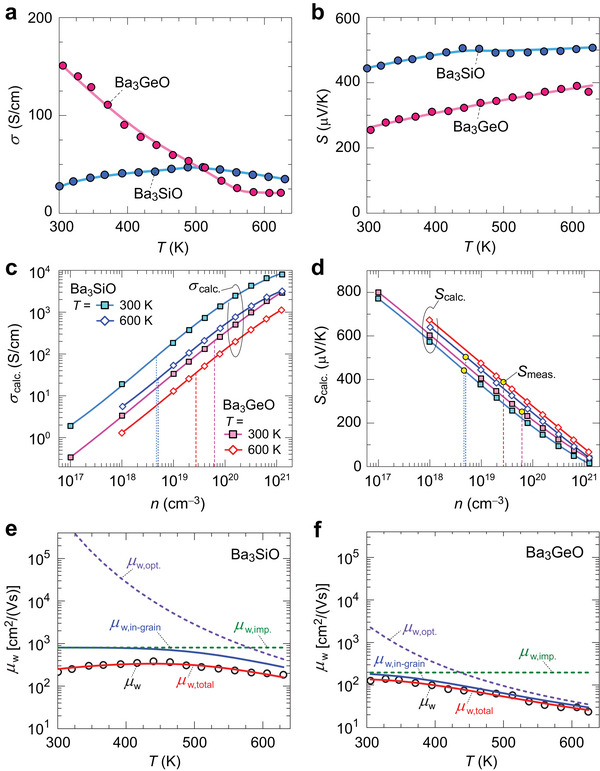
Carrier transport properties of Ba_3_ *B*O (*B* = Si and Ge). a,b) Temperature (*T*) dependences of a) electronic conductivity (*σ*) and b) Seebeck coefficient (*S*) of Ba_3_ *B*O bulks. c,d) Calculated electronic transport coefficients: c) *σ*_calc._ and d) *S*_calc._ as a function of carrier concentration (*n*) at *T* = 300 K and 600 K. The solid lines are the polynominal fitting to the data points of *σ*_calc._ vs *n* and *S*_calc._ vs *n*. The measured *S* (*S*_meas._) of Ba_3_ *B*O bulks are plotted in (d), and the dotted lines indicate the *n* estimated from the *S*_meas._ in the *S*_calc._ vs *n* relations. e,f) Weighted carrier mobility (*µ*_w_) vs. *T* plots fitted by $\mu_{w,\mathrm{total}}\left(T\right)=\exp\left(\frac{qE_{b}}{k_{B}T}\right)\mu_{w,\mathrm{in}-\mathrm{grain}}\left(T\right)$, where µ_w,in − grain_(*T*) is obtained by the Matthiessen's rule, $\mu_{w,\mathrm{in}-\mathrm{grain}}^{-1}\;=\mu_{w,\mathrm{imp}.}^{-1}+\mu_{w,\mathrm{opt}.}^{-1}$, for e) Ba_3_SiO bulk and f) Ba_3_SiO bulk. The red lines show the total mobility (*µ*_w,total_) and the blue lines show the in‐grain *µ*_w_ without GB scattering (*µ*_w,in‐grain_). The green and the purple dotted lines show the ionized impurity scattering limited mobility (*µ*_w,imp._) and the optical phonon scattering limited mobility (*µ*_w,opt._), respectively.

**Figure** **3**a,b shows the temperature (*T*) dependence of *σ* and *S* for Ba_3_ *B*O bulks. The Ba_3_GeO bulk exhibits higher *σ* = 151 S cm^−1^ than 28 S cm^−1^ of Ba_3_SiO bulk at *T* ≃ 300 K (Figure 3a). The metallic *T* dependence of *σ* is observed for the Ba_3_GeO bulk over the whole *T* range. On the other hand, *σ* of Ba_3_SiO bulk shows metallic *T* dependence at high *T* ≥ 510 K, while *σ* decreases at *T* < 500 K. Both the samples show positive *S* over the whole *T* range (Figure 3b), indicating the majority carrier is hole. The *S* = +444 µV K^−1^ for Ba_3_SiO is larger than +255 µV K^−1^ for Ba_3_GeO at *T* ≃ 300 K. The *S* value linearly increases to +507 µV K^−1^ at *T* = 630 K for Ba_3_SiO and +372 µV K^−1^ at *T* = 624 K for Ba_3_GeO with increasing *T*. Note that Hall effect measurement was difficult to perform because these samples are sensitive to air and sample degradation occurs during the transfer to the measurement system.

We then calculated the carrier lifetime (*τ*_e_) and obtained the carrier concentration (*n*) dependences of *σ*_calc._ and *S*_calc._ at *T* = 300 K and 600 K (Figure 3c,d) by density functional perturbation theory (DFPT)^[^ ^37^ ^]^ as implemented in Quantum ESPRESSO package^[^ ^38^, ^39^ ^]^ and PERTURBO code.^[^ ^40^ ^]^ It is seen that *σ*_calc._ increases while *S*_calc._ decreases with *n* as usually observed due to the well‐known competitive relationship. The *σ*_calc._ of Ba_3_SiO is nearly one order of magnitude higher than that of Ba_3_GeO, which originates from the smaller *τ*_e_ in Ba_3_GeO due to strong electron–phonon scattering (Figure S4, Supporting Information). Here, we need to compare *σ*_calc._ and the measured *σ* but *σ*_calc._ is a function of *n*. We, therefore, first estimated experimental *n* from the measured *S* (*S*_meas._) using the calculated *S*_calc._ vs *n* relation as a reference. We estimated *n* to be ≈4.8 × 10^18^ cm^−3^ (≈5.0 × 10^18^ cm^−3^) for the Ba_3_SiO bulk and ≈6.2 × 10^19^ cm^−3^ (≈2.7 × 10^19^ cm^−3^) for the Ba_3_GeO bulk at *T* = 300 K (600 K) as shown by the yellow circles in Figure 3d, indicating that the *n* of Ba_3_SiO is one order of magnitude lower than that of Ba_3_GeO. The *n* exhibits a weak *T* dependence for both the Ba_3_SiO and the Ba_3_GeO bulks, indicating degenerate hole conduction, which is supported by X‐ray photo‐emission spectroscopy spectra near the VBM (Figure S5, Supporting Information) because the Fermi level locates 0.1 eV below the VBM. We finally estimated *σ*_calc._ at the estimated *n* as the crossing points of the solid and dotted lines in Figure 3c.

**Table** **1** compares the experimentally measured and theoretically calculated carrier transport properties of Ba_3_ *B*O. Prior to explaining the detailed results, we like to summarize that the experimentally obtained results are consistent with the calculated ones both at *T* = 300 K and 600 K for Ba_3_GeO while three times differences are found at *T* = 300 K for Ba_3_SiO. For Ba_3_GeO, the *σ*_calc._ (*n* at 300 K) = 195 S cm^−1^ and *σ*_calc._ (*n* at 600 K) = 35 S cm^−1^ are almost consistent with the measured *σ* (*σ*_meas._) = 151 S cm^−1^ at 300 K and 21 S cm^−1^ at 600 K, respectively. Accordingly, carrier mobility µ = σ_meas._/*en* and $\tau_{e}=m_{\mathrm{band}}^{*}\mu /e$ ($m_{\mathrm{band}}^{*}\;$is band effective mass) in Table 1 show similar consistency. On the other hand, for Ba_3_SiO, although the *σ*_calc._ (*n* at 600 K) = 28 S cm^−1^ is consistent with *σ*_meas._ = 37 S cm^−1^ at *T* = 600 K, but *σ*_calc._ (*n* at 300 K) = 89 S cm^−1^ is three‐times higher than *σ*_meas._ = 28 S cm^−1^ at *T* = 300 K. Accordingly, the estimated *µ* = 46.6 cm^2^ V^−1^ s^−1^ is consistent with *µ*_calc._ = 35.0 cm^2^ V^−1^ s^−1^ at *T* = 600 K, while they show three‐times difference (*µ* = 36.4 cm^2^ V^−1^ s^−1^ and *µ*_calc._ = 115.7 cm^2^ V^−1^ s^−1^) at 300 K. We here need to recognize that the calculated results reflect the transport properties of the ideal single crystal while the experimental ones include carrier scattering by defects and grain boundaries (GB), giving the significant discrepancy at lower temperatures.

**Table 1:** Summary of experimentally measured and theoretically calculated carrier transport properties for Ba_3_ *B*O (*B* = Si and Ge) at *T* = 300 K and 600 K. $m_{\mathrm{band}}^{*}$ is the band effective mass and $m_{\mathrm{DOS}}^{*}$ is the density of state effective mass, calculated by BoltzTraP2 code. *n* is the carrier concentration obtained from the measured *S* in calculated *S*_calc._ vs *n* relation (Figure 3d). *σ*_meas._ is the experimentally measured electronic conductivity (Figure 3a). *σ*_calc._ is the calculated electronic conductivity obtained from the calculated *σ*_calc._ vs *n* relation at the estimated *n* (Figure 3c). *µ* and *µ*_calc._ are the carrier mobility obtained by µ = σ_meas._/*en* and µ_calc._ = σ_calc._/*en*. *τ*_e_ and *τ*_e,calc._ are the carrier life time obtained by $\tau_{e}=m_{\mathrm{band}}^{*}\mu /e$ and $\tau_{e,\mathrm{calc}.}=m_{\mathrm{band}}^{*}\mu_{\mathrm{calc}.}/e$.

	$m_{\mathrm{band}}^{*}$	$m_{\mathrm{DOS}}^{*}$	*T* [K]	*n* [cm^−3^]	*σ*_meas._ [S cm^−1^]	*σ*_calc._ [S cm^−1^]	*µ* [cm^2^ V^−1^ s^−1^]	*µ*_calc._ [cm^2^ V^−1^ s^−1^]	*τ*_e_ [fs]	*τ*_e,calc_.[fs]
Ba_3_SiO	0.85*m*_0_	2.46*m*_0_	300	4.8×10^18^	28	89	36.4	115.7	17.6	55.9
			600	5.0×10^18^	37	28	46.6	35.0	22.5	16.9
Ba_3_GeO	0.72*m*_0_	2.24*m*_0_	300	6.2×10^19^	151	195	15.2	19.6	6.2	8.0
			600	2.7×10^19^	21	35	4.8	8.1	2.0	3.3

To separate the single‐crystalline‐like carrier transport in crystalline grains and the effect of GBs, we employ the Seto model,^[^ ^42^ ^]^ $\mu \left(T\right)=\exp\left(\frac{qE_{b}}{k_{B}T}\right)\mu_{\mathrm{in}-\mathrm{grain}}\left(T\right)$, where µ_in − grain_(*T*) is the in‐grain carrier mobility and the GB contribution is expressed as $\exp(\frac{qE_{b}}{k_{B}T})$ (*E*_b_ is the GB barrier height and *k*_B_ is the Boltzmann constant). As we could not perform Hall effect measurements, we estimate µ(*T*) as weighted mobility from σ and *S* by $\mu_{w}=\frac{3h^{3}\sigma }{8\pi e{\left(2m_{e}k_{B}T\right)}^{3/2}}\left[\frac{exp[\frac{\left|S\right|}{k_{B}/e}-2]}{1+exp[-5(\frac{\left|S\right|}{k_{B}/e}-1)]}+\frac{\frac{3}{\pi^{2}}\;\frac{\left|S\right|}{k_{B}/e}}{1+exp[5(\frac{\left|S\right|}{k_{B}/e}-1)]}\right]$, where *m*_e_ is the free electron mass.^[^ ^41^ ^]^ The *µ*_w_ is related to the drift mobility *µ* by $\mu_{w}\approx \mu {\left(\frac{m_{\mathrm{DOS}}^{*}}{m_{e}}\right)}^{3/2}$, where $m_{\mathrm{DOS}}^{*}$ is density of state effective mass. The *µ*_w_ of Ba_3_SiO bulk exhibits a negative *T* coefficient at *T* ≥ 440 K, while it decreases with a decrease of *T* at low *T* region ≤ 440 K (the black circles in Figure 3e). The *µ*_w_ of Ba_3_GeO bulk increases with a decrease of *T* in a wide range of *T* ≥ 327 K but it levels off at *T* < 327 K (the black circles in Figure 3f). Then, µ_w,in − grain_(*T*) is modeled by Matthiessen's rule, $\mu_{w,\mathrm{in}-\mathrm{grain}}^{-1}\;=\mu_{w,\mathrm{imp}.}^{-1}+\mu_{w,\mathrm{opt}.}^{-1}$. The impurity scattering mobility is expressed as $\mu_{w,\mathrm{imp}.}^{-1}=A$ (temperature independent) in the degenerate regime. The optical phonon scattering mobility is expressed as $\mu_{w,\mathrm{opt}.}^{-1}=1/\;\left(B(\exp(\frac{\hbar \omega_{0}}{kT})-1)\right)$ (B is a constant), where ℏω_0_ is the longitudinal optical phonon energy. These parameters are obtained by least‐squares fitting of the total mobility $\mu_{w,\mathrm{total}}\left(T\right)=\exp\left(\frac{qE_{b}}{k_{B}T}\right)\mu_{w,\mathrm{in}-\mathrm{grain}}\left(T\right)$ to the experimental µ_w_(*T*). In Figure 3e,f, the solid red line shows the *µ*_w,total_ providing good agreement with experimental *µ*_w_ over a wide *T* range. At high *T* region, the *µ*_w,in‐grain_ (blue lines) is dominated by optical phonon scattering (the purple dotted lines), where the ℏω_0_ values were optimized to be 260 meV for Ba_3_SiO and 210 meV for Ba_3_GeO. The *µ*_w,in‐grain_ increases and approaches the *µ*_w,imp._ (green dotted lines), when *T* is reduced to ≈300 K. The *µ*_w,in‐grain_ is higher than *µ*_w,total_ especially at lower *T* range for Ba_3_SiO bulk, indicating that the carrier transport is limited by GB scattering. The *µ*_w,in‐grain_ is 3.2 times higher than *µ*_w,total_ at *T* ≃ 300 K, which is consistent with the *µ*_calc._/*µ* = 3.2 obtained in Table 1. For Ba_3_GeO, the *µ*_w,in‐grain_ is nearly the same with *µ*_w,total_ in a wide range of *T* ≥ 327 K but the difference becomes a little larger at *T* < 327 K. The *µ*_w,in‐grain_ is 1.3 times higher than *µ*_w,total_ at *T* ≃ 300 K, in consistence with the *µ*_calc._/*µ* = 1.3. The *E*_b_ is estimated to be 28 meV for Ba_3_SiO while *E*_b_ = 8 meV is much smaller for Ba_3_GeO. The higher density carriers in Ba_3_GeO bulk may screen the GB background charges and reduce the GB barrier heights. Although the Ba_3_ *B*O bulks have a relatively poor polycrystalline nature with low sintered densities 80–87% and the carrier transport of Ba_3_SiO bulk is limited by GB scattering at low *T* region, the mobility analysis suggests that Ba_3_ *B*O possess potentially high carrier mobility.

Next, effective masses *m* ^*^ are estimated as *m* ^*^ determines *S* in the simple free electron model by $S=\frac{k_{B}}{e}\left(\frac{3}{2}\ln m_{\mathrm{DOS}}^{*}+\ln2{\left(\frac{2\pi k_{B}T}{h^{2}}\right)}^{\frac{3}{2}}+r+2-\ln n\right)$, where $m_{\mathrm{DOS}}^{*}$ is the density‐of‐states effective mass at VBM. Here the band effective masses ($m_{\mathrm{band}}^{*}$= $Ne^{2}\frac{\tau_{e}}{\sigma }$) at VBM are calculated to be slightly large at 0.85*m*_0_ for Ba_3_SiO and 0.72*m*_0_ for Ba_3_GeO (Table 1). However, the calculated carrier lifetime (τ_e,calc._) is long at 55.9 fs for Ba_3_SiO and 8.0 fs for Ba_3_GeO, resulting in the relatively high *µ*_calc._ of 115.7 and 19.6 cm^2^ V^−1^ s^−1^ at *T* = 300 K, respectively. The dispersive bands at VBM with relatively small $m_{\mathrm{band}}^{*}$ and long τ_e_ contribute to high *µ* ($=\frac{e\tau_{e}}{m_{\mathrm{band}}^{*}}$) and *σ* (= µ*ne*). However, the lifetime calculations were performed at the rigid band scheme (no ion dynamics) and polaron effect is not considered but it can reduce the real *µ*. On the other hand, $m_{\mathrm{DOS}}^{*}$ are calculated to be 2.46*m*_0_ for Ba_3_SiO and 2.24*m*_0_ for Ba_3_GeO. The large $m_{\mathrm{DOS}}^{*}$ originates from the high valence band degeneracy as explained for Figure 2b,c, which contributes to the large *S*. Therefore, the negatively‐charged *B* ion contributes to hole transport with long carrier life time, and the dispersive valence bands (small $m_{\mathrm{band}}^{*}$) with the high valley degeneracy (large *m*_DOS_^*^) are suitable for realizing high PF (= *S* ^2^ *σ*).

### 2.3 Thermal Transport Properties

**Figure 4:**
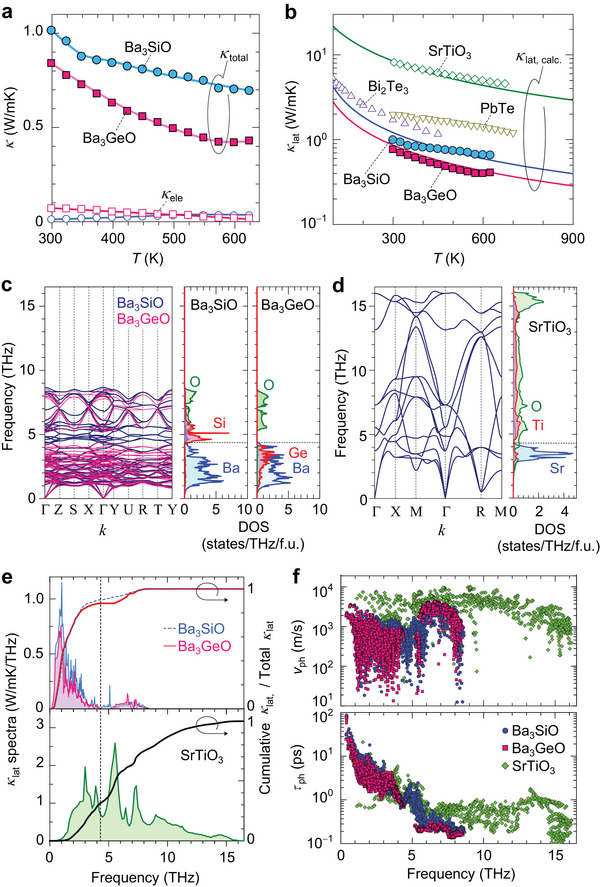
Phonon transport properties of Ba_3_ *B*O (*B* = Si and Ge). a) Temperature (*T*) dependences of total thermal conductivity (*κ*_total_) and electronic thermal conductivity (*κ*_ele_) for Ba_3_ *B*O bulks. b) *T* dependence of lattice thermal conductivity (*κ*_lat_) for Ba_3_ *B*O bulks, compared with the reported *κ*_lat_ of normal perovskite SrTiO_3_ bulk^[^ ^44^ ^]^ as well as PbTe and Bi_2_Te_3_ bulks.^[^ ^45^, ^46^, ^47^ ^]^ Calculated *κ*_lat_ of Ba_3_ *B*O and SrTiO_3_ models are also shown by the solid lines. c,d) Anharmonic phonon dispersions (left panel) and partial phonon density of states (DOSs) projected on each element (right panel) for c) Ba_3_ *B*O and d) SrTiO_3_ at *T* = 300 K obtained by the self‐consistent phonon (SCPH) approximation. e) Comparison of *κ*_lat_ spectra for Ba_3_ *B*O (top panel) and SrTiO_3_ (bottom panel) at *T* = 300 K. Frequency‐dependent cumulative *κ*_lat_ normalized by total *κ*_lat_ is also shown for each panel. f) Phonon group velocity, *ν*_ph_ (top panel), and phonon lifetime, *τ*_ph_ (bottom panel) in terms of the phonon frequency.

Next we discuss thermal transport properties by separating electronic and lattice contributions. **Figure** **4**a summarizes the *T* dependence of total *κ* (*κ*_total_) and electronic *κ* (*κ*_ele_) of Ba_3_SiO and Ba_3_GeO bulks. The *κ*_ele_ is calculated by Wiedemann‐Franz law as *κ*_ele_ = *LTσ*, where *L* is the Lorenz number calculated from $L={\left(\frac{k_{B}}{e}\right)}^{2}\left(\frac{\left(r+\frac{7}{2}\right)F_{r+5/2}\left(\eta \right)}{\left(r+\frac{3}{2}\right)F_{r+\frac{1}{2}}\left(\eta \right)}-{\left[\frac{\left(r+\frac{5}{2}\right)F_{r+3/2}\left(\eta \right)}{\left(r+\frac{3}{2}\right)F_{r+1/2}\left(\eta \right)}\right]}^{2}\right)$. Here, the reduced Fermi energy *η* is obtained based on the free carrier model using the measured *S* as $S=\frac{k_{B}}{e}\left(\frac{\left(r+\frac{5}{2}\right)F_{r+3/2}\left(\eta \right)}{\left(r+\frac{3}{2}\right)F_{r+1/2}\left(\eta \right)}-\eta \right)$ with the Fermi integral defined as $F_{n}\left(\eta \right)=\int_{0}^{\infty }\frac{\chi^{n}}{1+e^{\chi -\eta }}d\chi$, where *γ* = −1/2 is the scattering factor.^[^ ^43^ ^]^ The Ba_3_SiO and Ba_3_GeO bulks showed low *κ*_total_ of 1.02 W m^−1^K^−1^ for Ba_3_SiO and 0.84 W m^−1^K^−1^ for Ba_3_GeO at *T* = 300 K. The *κ*_total_ values of Ba_3_SiO and Ba_3_GeO bulks decrease to 0.69 W m^−1^K^−1^ and 0.43 W m^−1^K^−1^ as the *T* rises to 623 K. The estimated *κ*_ele_ was less than 0.1 W m^−1^K^−1^, where the maximum *κ*_ele_ was 0.04 W m^−1^K^−1^ at *T* = 523 K for Ba_3_SiO and 0.07 W m^−1^K^−1^ at *T* = 300 K for Ba_3_GeO, indicating the small electronic contribution to *κ*_total_. Then, the lattice *κ* (*κ*_lat_) is extracted by subtracting the electronic contribution from the *κ*_total_, i.e. *κ*_lat_ = *κ*_total_ – *κ*_ele_. The *T* dependences of *κ*_lat_ are summarized in  Figure 4b, where those of the normal perovskite SrTiO_3_ bulk^[^ ^44^ ^]^ as well as representative chalcogenides of Bi_2_Te_3_ and PbTe bulks^[^ ^45^, ^46^, ^47^ ^]^ are superimposed for comparison. The *κ*_lat_ decreases from 1.00 W m^−1^ K^−1^ at *T* = 300 K to 0.66 W m^−1^ K^−1^ at *T* = 623 K for Ba_3_SiO and it decreases from 0.77 W m^−1^ K^−1^ at RT to 0.41 W m^−1^ K^−1^ at *T* = 623 K for Ba_3_GeO. The *κ*_lat_ at *T* = 300 K is much lower than 8.2 W m^−1^ K^−1^ of SrTiO_3_ bulk^[^ ^44^ ^]^ and also even lower than ≈1.7 W m^−1^ K^−1^ of Bi_2_Te_3_ bulk^[^ ^45^ ^]^ and ≈2.0 W m^−1^ K^−1^ of PbTe bulk,^[^ ^47^ ^]^ while it is comparable to *κ*_lat_ of state‐of‐the‐art chalcogenide thermoelectric materials, such as 0.7 W m^−1^K^−1^ of SnSe bulk,^[^ ^48^ ^]^ 0.6 W m^−1^K^−1^ of Cu_2_Se bulk,^[^ ^49^ ^]^ and 0.6–0.8 W m^−1^K^−1^ of GeTe bulk.^[^ ^7^ ^]^

### 2.4 Origin of Low Lattice Thermal Conductivity in Inverse‐Perovskite

We compared the phonon transport properties of Ba_3_ *B*O with the normal perovskite SrTiO_3_ to discern the distinguishing characteristics of inverse perovskites. First, we performed the simple phonon gas model analysis using $\kappa_{\mathrm{lat}}=\frac{1}{3}C_{v}\cdot v_{s}\cdot l_{\mathrm{ph}}=\frac{1}{3}C_{v}\cdot v_{s}^{2}\cdot \tau_{\mathrm{ph}}$, where *C*_v_ is the specific heat per volume, *v*_s_ is the sound velocity, *l*_ph_ is the phonon mean free path, and *τ*_ph_ is the phonon lifetime (Table S1, Supporting Information). The *v*_s_, measured by ultrasonic pulse echo method at RT, are 2317 m s^−1^ (1981 m s^−1^) for Ba_3_SiO (Ba_3_GeO), which is less than a half of 5241 m s^−1^ for the normal perovskite SrTiO_3_. In addition, the estimated *τ*_ph_ of 0.16 ps (0.16 ps) for Ba_3_SiO (Ba_3_GeO) is a half of 0.32 ps of SrTiO_3_. The smaller *v*_s_ and lower *τ*_ph_ lead to the shorter *l*_ph_ (= *v*_s_ *τ*_ph_) of 0.38 nm (0.32 nm) for Ba_3_SiO (Ba_3_GeO) than 1.70 nm for SrTiO_3_, resulting in the low *κ*_lat_. The bulk modulus was calculated to be 92.8 GPa (80.0 GPa) and the Debye temperature was 220 K (187 K) for Ba_3_SiO (Ba_3_GeO). The estimated Grüneisen parameter was relatively large at 1.3–1.4, which is comparable to low *κ*_lat_ thermoelectric materials such as Bi_2_Te_3_ and PbTe,^[^ ^50^ ^]^ Therefore, both the low sound velocity and the strong phonon scattering are responsible for the intrinsically low *κ*_lat_ in Ba_3_ *B*O.

To further elucidate the underlying mechanism responsible for the low *κ*_lat_ in Ba_3_ *B*O, we conducted first‐principles anharmonic lattice dynamics (ALD) calculations based on the density functional theory (DFT), as implemented in the ALAMODE code.^[^ ^52^, ^53^ ^]^ Figure 4c,d compares the anharmonic phonon dispersions (left panels) and the partial phonon DOSs projected on each element (right panels) for Ba_3_ *B*O and SrTiO_3_ models at *T* = 300 K. Ba_3_ *B*O exhibits flatter phonon bands and all phonon bands only exist at a low frequency below 9 THz (left panel of Figure 4c). The phonon DOSs of the Ba_3_SiO reveal that the vibrations of Ba atoms predominantly contribute to the lower frequency phonon bands with the cut‐off frequency ≈4.3 THz, while the Si and O atoms primarily contribute to higher frequency phonon branches (right panel of Figure 4c). For Ba_3_GeO, the phonon bands of heavier Ge ion shift to lower frequency and have interaction with the phonon bands of Ba ion. On the other hand, SrTiO_3_ exhibits largely dispersive phonon bands even at higher frequencies (left panel of Figure 4d), with extensive dispersion at high frequencies of 4–14 THz, originating from the cooperative Ti and O atomic vibration (right panel of Figure 4d). The dispersion is considerably larger than observed in the low‐frequency phonon bands primarily attributed to Sr atomic vibrations at frequencies below ≈4.3 THz.

We then calculated *κ*_lat_ by solving the Peierls–Boltzmann transport equation (PBTE) within the relaxation time approximation. The calculated *κ*_lat_ (averaged along *a*,*b*,*c*‐axes) as a function of *T* for Ba_3_ *B*O and SrTiO_3_ are compared in Figure 4b. The calculated *κ*_lat_ of Ba_3_SiO (Ba_3_GeO) are 1.21 W m^−1^ K^−1^ (0.86 W m^−1^ K^−1^) and that of SrTiO_3_ is 8.46 W m^−1^ K^−1^ at *T* = 300 K, in consistence with the experimentally measured values. Figure 4e compares the *κ*_lat_ spectra at *T* = 300 K with respect to phonon frequency for Ba_3_ *B*O and SrTiO_3_ models. The frequency‐dependent cumulative *κ*_lat_ normalized by total *κ*_lat_ are also shown in the panels. For Ba_3_ *B*O, the *κ*_lat_ spectra peak at ≈1 THz and phonons in the low‐frequency region below ≈4.3 THz contribute mostly to *κ*_lat_. The acoustic and optical modes are hybridized when the *q* point is far from the Γ point, making it difficult to distinguish the acoustic and optical contributions clearly. However, if we consider a cut‐off frequency for acoustic phonons, at which the acoustic and optical branches cross (Figure 4c), at ≈1.4 THz for Ba_3_ *B*O, the contribution of low‐frequency acoustic phonons to *κ*_lat_ is ≈55%. The low‐frequency acoustic phonons and mid‐frequency optical phonons within 4.3 THz contribute to the ≈91% of total *κ*_lat_, indicating that heat conduction primarily arises from the vibrational motion of Ba atoms (also Ge atoms in Ba_3_GeO). In contrast, for SrTiO_3_, the low‐frequency acoustic phonons and mid‐frequency optical phonons within 4.3 THz contribute to only the ≈31% of total *κ*_lat_, i.e., not only the low‐frequency Sr atom vibrations but also high‐frequency phonons associated with Ti and O atomic vibrations contribute greatly to heat conduction.

Figure 4f compares the phonon group velocity, *ν*_ph_ (top panel) and phonon lifetime, *τ*_ph_ (bottom panel) in terms of the phonon frequency. Ba_3_ *B*O exhibit much lower *ν*_ph_ than SrTiO_3_ across all frequency ranges, primarily due to the presence of flat phonon band branches (left panel of Figure 4c). On the other hand, *τ*_ph_ is almost similar at low frequency (<3.5 THz) for both Ba_3_ *B*O and SrTiO_3_, but the value of Ba_3_ *B*O becomes smaller at higher frequency region (3.5–5 THz). For SrTiO_3_, the *τ*_ph_ becomes smaller at high frequency (>6 THz), but *ν*_ph_ is still large, reflecting the widely spread optical phonon bands. Therefore, the large *ν*_ph_ for phonons associated not only with Sr atomic vibration but also with Ti and O atomic vibrations provides a large contribution to high *κ*_lat_ in SrTiO_3_. The *τ*_ph_ of Ba_3_ *B*O becomes further smaller in the higher frequency region. The low *κ*_lat_ of Ba_3_ *B*O predominantly originates from the low *ν*_ph_ for phonons associated with Ba atomic vibration, and the phonons associated with Si and O atomic vibrations have a negligible contribution to *κ*_lat_ due to the very short *τ*_ph_. Ba_3_ *B*O shows more phonon bands than SrTiO_3_ (left panel of Figure 4c), because it has the local structure distortion with lower crystalline symmetry, resulting in the splitting of degenerated phonon bands. These broad frequency shifts enhance the phonon‐phonon scattering probability that leads to a large *τ*_ph_ reduction in Ba_3_ *B*O. The inverse and the normal perovskite structures are constructed from the network of O−Ba_6_ and Ti−O_6_ octahedra. Then, the bonding energies of O−Ba in Ba_3_ *B*O and Ti−O in SrTiO_3_ as a measure of bonding strengths were estimated through the chemical bonding analysis using the crystal orbital Hamilton population (COHP)^[^ ^53^ ^]^ performed by the LOBSTER code.^[^ ^54^ ^]^ For the Ba_3_ *B*O case, the −iCOHP values (the integrated −COHP up to the Fermi level, corresponding to the bond strength) averaged for O−Ba bonds are as small as 0.276 eV per bond for Ba_3_SiO and 0.283 eV per bond for Ba_3_GeO, indicating ionic interaction between the Ba atom and O atom in O−Ba_6_ octahedra of the Ba_3_ *B*O. On the other hand, the Ti−O bonds of SrTiO_3_ have more than 10 times larger −iCOHP values of 3.48 eV per bond, originating from the strong covalent interaction between the Ti atom and the O atom in SrTiO_3_ lattice. The inverse‐perovskite Ba_3_ *B*O has a similar crystal structure to the perovskite SrTiO_3_, but the ionic nature of the O−Ba bond softens the octahedra framework and thus the contribution of high‐frequency optical phonons is negligible for heat transport in Ba_3_ *B*O. Note that the related phenomenon of low *κ*_lat_ and strong phonon scattering is observed in layered BaAgSb with weakly ionic bonded Ba atoms.^[^ ^55^ ^]^

### 2.5 Thermoelectric Properties

**Figure 5:**
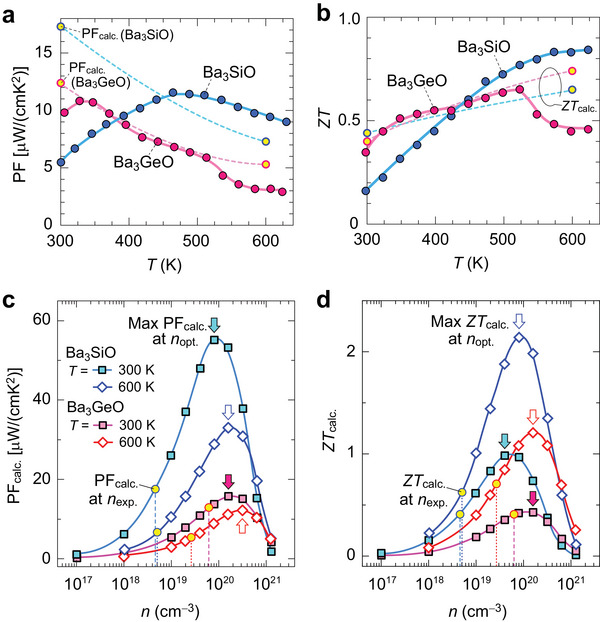
Thermoelectric properties of Ba_3_ *B*O (*B* = Si and Ge). a,b) Temperature (*T*) dependences of a) power factor (PF) and b) dimensionless figure of merit (*ZT*) of Ba_3_ *B*O bulks. c,d) Carrier concentration (*n*) dependences of c) calculated PF (PF_calc._ = *S* ^2^ *σ*_calc._) and d) calculated *ZT* (*ZT*_calc._ = *S* ^2^ *σ*_calc._ *T*/*κ*_calc._) at *T* = 300 K and 600 K. The arrows indicate the maximum PF_calc._ and *ZT*_calc._ at the optimal *n* (*n*_opt._). The yellow circles in a‐d indicate the PF_calc._ and *ZT*_calc._ obtained from the experimental *n* (*n*_exp._) estimated in Figure 3d.

**Figure** **5**a,b summarizes the *T* dependences of PF (= *S* ^2^ *σ*) and *ZT* (= *S* ^2^ *σT*/*κ*) of the Ba_3_ *B*O bulks. The calculated PF (PF_calc._ = *S* ^2^ *σ*_calc._) and *ZT* (*ZT*_calc._ = *S* ^2^ *σ*_calc._ *T*/*κ*_calc._) are also superimposed in the panel. The PF of Ba_3_SiO and Ba_3_GeO bulks are limited to 5.5 and 9.8 µW cm^−1^ K^−2^ at *T* ≃ 300 K, respectively, because of GB scattering (Figure 5a), but, as a consequence of their low *κ*, Ba_3_SiO and Ba_3_GeO show relatively high *ZT* = 0.16 and 0.35 at *T* ≃ 300 K, respectively (Figure 5b). On the other hand, they potentially show higher PF_calc._ = 17.3 and 12.4 µW cm^−1^ K^−2^ by eliminating GB scattering, and *ZT*_calc._ would be increased up to 0.44 and 0.40 for Ba_3_SiO and Ba_3_GeO, respectively. The PF of Ba_3_SiO largely increases to 11.6 µW cm^−1^ K^−2^ when *T* increases to 464 K, and then decreases to 9.0 µW cm^−1^ K^−2^ at high *T* = 630 K. The *ZT* value increases continuously up to 0.84 at *T* = 623 K, which is slightly higher than *ZT*_calc._ = 0.65 at *T* = 600 K due to higher *σ* than *σ*_calc._ On the other hand, Ba_3_GeO exhibits the maximum PF = 10.8 µW cm^−1^ K^−2^ at low *T* = 327 K, and the PF decreases continuously down to 5.9 µW cm^−1^ K^−2^ at *T* = 523 K, where the maximum *ZT* = 0.65 was obtained. The *ZT*_calc._ of Ba_3_GeO increases continuously up to 0.74 at *T* = 600 K, but the PF and *ZT* suddenly decrease at *T* ≥ 548 K due to the decrease of *σ* (Figure 3a) and the increase of *κ* (Figure 4a). We speculate that these *σ* and *κ* changes of Ba_3_GeO would originate from the transition to higher symmetric inverse‐perovskite structure at high *T* because it has slightly high tolerance factor = 0.918. The high *T* crystal‐structure characterization is necessary for this conclusion.

**Figure 6:**
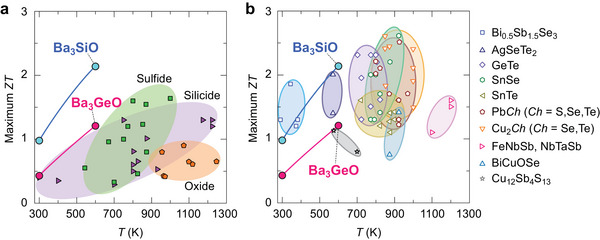
Comparison of maximum *ZT* as a function of temperature (*T*) for Ba_3_ *B*O (*B* = Si and Ge) with respect to a) eco‐friendly thermoelectric materials including sulfides (Cu_2_S,^[^ ^56^, ^57^, ^58^, ^59^ ^]^ SnS,^[^ ^60^ ^]^ Cu_0.1_TiS_2_,^[^ ^61^ ^]^ (Cu,Fe)S_2_,^[^ ^62^ ^]^ Cu_7_Sn_3_S_10_,^[^ ^63^ ^]^ Cu_5_FeS_4_,^[^ ^64^ ^]^ Cu_26_Ta_2_Sn_5.5_S_32_,^[^ ^65^ ^]^ Cu_2_ZnSnS_4_^[^ ^66^ ^]^), silicides (Si_0.8_Ge_0.2_,^[^ ^67^ ^]^ Mg_2_Si,^[^ ^68^, ^69^, ^70^ ^]^ Mg_2_(Si,Sn),^[^ ^71^ ^]^ MnSi_1.75_,^[^ ^72^ ^]^ CrSi_2_,^[^ ^73^ ^]^ β‐FeSi_2_,^[^ ^74^ ^]^ Sr_0.92_Y_0.08_Si_2_^[^ ^75^ ^]^), oxides (Sr_0.9_La_0.1_Ti_0.9_Nb_0.1_O_3_,^[^ ^76^ ^]^ Sr_0.93_La_0.07_Ti_0.93_Nb_0.07_O_3_,^[^ ^77^ ^]^ Sr_0.775_La_0.15_□_0.075_TiO_3−_ *_δ_*,^[^ ^78^ ^]^ Zn_0.96_Al_0.02_Ga_0.02_O,^[^ ^79^ ^]^ Na_1.7_Co_2_O_4_,^[^ ^80^ ^]^ Ca_2.8_Ag_0.05_Lu_0.15_Co_4_O_9+_ *_δ_*,^[^ ^81^ ^]^ Ca_2.95_Tb_0.05_Co_4_O_9_Bi_0.25_^[^ ^82^ ^]^), and b) state‐of‐the‐art thermoelectric materials with heavy (toxic) elements: Bi_0.5_Sb_1.5_Se_3_,^[^ ^83^, ^84^, ^85^, ^86^ ^]^ AgSeTe_2_,^[^ ^87^, ^88^ ^]^ GeTe,^[^ ^89^, ^90^, ^91^, ^92^, ^93^, ^94^, ^95^, ^96^ ^]^ SnSe,^[^ ^97^, ^98^, ^99^, ^100^, ^101^, ^102^, ^103^, ^104^ ^]^ SnTe,^[^ ^105^, ^106^, ^107^, ^108^, ^109^, ^110^, ^111^, ^112^ ^]^ Pb*Ch* (*Ch* = S, Se, Te),^[^ ^113^, ^114^, ^115^, ^116^, ^117^, ^118^, ^119^, ^120^, ^121^ ^]^ Cu_2_ *Ch* (*Ch* = Se, Te),^[^ ^49^, ^122^, ^123^, ^124^, ^125^, ^126^, ^127^, ^128^, ^129^ ^]^ Half Heusler FeNbSb and FeTaSb,^[^ ^130^, ^131^, ^132^ ^]^ BiCuOSe,^[^ ^133^, ^134^ ^]^ Tetrahedrite Cu_12_Sb_4_S_13_.^[^ ^135^, ^136^ ^]^ Green, purple, and orange symbols in (a) indicate maximum *ZT* values for sulfide, silicide, and oxide thermoelectric materials, respectively.

Note that we tried hole doping to obtain optimum *ZT* by potassium ion (K^+^) substitution for Ba_3_SiO. The *σ* is increased by K doping and the two‐orders of magnitude increase of carrier concentration is observed in (Ba_2.6_K_0.4_)SiO. However, a considerably large amount of K dopant is necessary to increase the carrier concentration and also carrier mobility is largely suppressed by such heavy K doping. Therefore, further exploration of efficient acceptor dopants is necessary to optimize their *ZT*. Instead, we estimate the maximum *ZT* of Ba_3_ *B*O with optimized *n* theoretically. We here calculate PF_calc._ and *ZT*_calc._ as a function of *n* (Figure 5c,d). The PF_calc._ vs *n* and *ZT*_calc._ vs *n* plots have maxima with respect to *n*, and the maximum values (max PF_calc._ and max *ZT*_calc._) are obtained at optimal *n* (*n*_opt._) as indicated by the arrows in Figure 5c,d. The *n*_opt._ are estimated to be 4.0 (8.1) × 10^19^ cm^−3^ and 1.6 (1.6) × 10^20^ cm^−3^ for Ba_3_SiO and Ba_3_GeO at *T* = 300(600) K. **Table** **2** summarizes the theoretical thermoelectric properties of Ba_3_ *B*O with *n*_opt._ at *T* = 300 and 600 K. The max *ZT*_calc._ of Ba_3_SiO is predicted to be 0.98 and 2.14 at *T* = 300 and 600 K, respectively, where the max PF_calc._ are much increased to 48.0 and 28.9 µW cm^−1^ K^−2^ by tuning *n* to *n*_opt._. The max *ZT*_calc._ of Ba_3_GeO are predicted to be 0.43 and 1.21 at *T* = 300 K and 600 K, respectively, where the max PF_calc._ is increased to 15.8 and 11.2 µW cm^−1^ K^−2^. The higher *ZT* of Ba_3_SiO compared with Ba_3_GeO accounts for its higher PF. **Figure** **6** compares the present maximum predictions for Ba_3_SiO and Ba_3_GeO with state‐of‐the‐art thermoelectric materials. The max *ZT*_calc._ value of Ba_3_SiO is much higher than those of eco‐friendly thermoelectric materials^[^ ^56^, ^57^, ^58^, ^59^, ^60^, ^61^, ^62^, ^63^, ^64^, ^65^, ^66^, ^67^, ^68^, ^69^, ^70^, ^71^, ^72^, ^73^, ^74^, ^75^, ^76^, ^77^, ^78^, ^79^, ^80^, ^81^, ^82^ ^]^ as seen in Figure 6a. Although higher *ZT* has been reported for the thermoelectric materials with heavy toxic elements of Pb, Te, Se, and Sb,^[^ ^49^, ^83^, ^84^, ^85^, ^86^, ^87^, ^88^, ^89^, ^90^, ^91^, ^92^, ^93^, ^94^, ^95^, ^96^, ^97^, ^98^, ^99^, ^100^, ^101^, ^102^, ^103^, ^104^, ^105^, ^106^, ^107^, ^108^, ^109^, ^110^, ^111^, ^112^, ^113^, ^114^, ^115^, ^116^, ^117^, ^118^, ^119^, ^120^, ^121^, ^122^, ^123^, ^124^, ^125^, ^126^, ^127^, ^128^, ^129^, ^130^, ^131^, ^132^, ^133^, ^134^, ^135^, ^136^ ^]^ the max *ZT*_calc._ value of Ba_3_SiO is comparable in the same temperature range (Figure 6b). For fair discussion, we like to note that these predictions are of ideal single crystals so the real maximum values would be reduced a bit by the impurity doping and consequent electron scattering. The present results, nonetheless, demonstrate the potential of inverse‐perovskite Ba_3_ *B*O as a high‐performance environmentally benign thermoelectric material that can be alternative to currently practical ones composed of heavy and toxic elements.

**Table 2:** Summary of theoretical thermoelectric properties for Ba_3_ *B*O (*B* = Si and Ge) with optimal *n* (*n*_opt._) at *T* = 300 K and 600 K.

	*T* [K]	*n*_opt._ [cm^−3^]	*σ*_calc._ [S cm^−1^]	*S*_calc._ [µV K^−1^]	PF_calc._ [µW cm^−1^ K^−2^]	*κ*_calc._ [W m^−1^ K^−1^]	*κ*_ele,calc._ [W m^−1^ K^−1^]	*κ*_lat,calc._ [W m^−1^ K^−1^]	*ZT*_calc._
Ba_3_SiO	300	4.0×10^19^	726.7	+257	48.0	1.46	0.25	1.21	0.98
	600	8.1×10^19^	411.2	+265	28.9	0.81	0.21	0.60	2.14
Ba_3_GeO	300	1.6×10^20^	503.5	+177	15.8	0.86	0.25	0.86	0.43
	600	1.6×10^20^	197.2	+238	11.2	0.43	0.13	0.43	1.21

## 3 Conclusion

In summary, we demonstrated the high *ZT* in bulk polycrystals of the p‐type inverse‐perovskite Ba_3_ *B*O (*B* = Si and Ge) without toxic elements. The valence band around the Fermi level arises from the *p* state of the negatively charged *B* anion with large ion size, and the hole transport with long carrier life time and their highly dispersive bands with multiple valley degeneracy realize both high *σ* and high *S*, simultaneously. In addition, the bulks exhibited low *κ*_lat_ of 1.00 W m^−1^ K^−1^ for Ba_3_SiO and 0.77 W m^−1^ K^−1^ for Ba_3_GeO at RT, which is significantly lower than 8.2 W m^−1^ K^−1^ of normal perovskite SrTiO_3_ bulk,^[^ ^44^ ^]^ and even lower than 1.7−2.0 W m^−1^ K^−1^ of Bi_2_Te_3_ and PbTe bulks.^[^ ^45^, ^46^, ^47^ ^]^ The low *κ*_lat_ of Ba_3_ *B*O originates from the low *ν*_ph_ for phonons associated with Ba atomic vibration, and the phonons associated with Si and O atomic vibrations have a negligible contribution to *κ*_lat_ due to the very short *τ*_ph_. The crystal structure of Ba_3_ *B*O is constructed from the highly distorted O−Ba_6_ octahedra framework with weak O−Ba ionic bonds, which provides extremely low *ν*_ph_ and strong phonon scattering. As a consequence of high PF and low *κ*_lat_, the Ba_3_SiO and Ba_3_GeO exhibited rather high *ZT* of 0.16 and 0.35 at RT, respectively. The *ZT* value increased continuously up to 0.84 at *T* = 623 K for Ba_3_SiO and 0.65 at *T* = 523 K for Ba_3_GeO. In addition, based on first‐principles carrier and phonon transport calculations, we predicted that a higher *ZT* could be obtained by optimizing hole concentration in Ba_3_ *B*O. Specifically, the maximum *ZT* potentially increases to 2.14 for Ba_3_SiO and 1.21 for Ba_3_GeO at *T* = 600 K. The present results indicate that inverse‐perovskites would be a new platform of environmentally benign high *ZT* thermoelectric materials.

## 4 Experimental Section

### Bulk Synthesis

The Ba_3_SiO and Ba_3_GeO bulk polycrystals were synthesized by solid‐state reactions of a stoichiometric mixture of Ba, Si or Ge, and BaO via a reaction of 2Ba + Si(Ge) +BaO → Ba_3_SiO(Ba_3_GeO). First, fresh Ba metal (purity 99.99%, Sigma–Aldrich) was finely cut into small pieces of grains.^[^ ^137^ ^]^ The Ba grain, Si (purity 99.9%, Kojundo Chemical Lab.) or Ge powders (purity 99.9%, Kanto Chemical), and BaO powder (purity 99.9%, Kanto Chemical) were mixed and then pressed into 10‐mm*ϕ* pellet. The obtained pellet was wrapped in Ta foil and then sealed in an Ar‐filled stainless tube. The sealed stainless tube was heated at an optimized temperature of 750 °C for 10 h for Ba_3_SiO and 700 °C for 10 h for Ba_3_GeO. The product was reground and densified to 10‐mmϕ pellet again, and then it was wrapped in Ta foil and then sealed in an Ar‐filled stainless tube. The sealed stainless tube was heated again at 900 °C for 10 h for Ba_3_SiO and 700 °C for 10 h for Ba_3_GeO. The bulk densities are 4.30 g cm^−3^ for Ba_3_SiO and 4.35 g cm^−3^ for Ba_3_GeO. The sintered densities are estimated to be 87.0% and 80.4%, respectively. The chemical compositions of the bulk samples measured with EDS are Ba_3.2_SiO_1.6_ and Ba_3.3_GeO_1.5_. The deviation from the stoichiometric composition would come from the coexistence of impurity phases, such as BaO, and the oxidation during sample transfer to measurement chamber. All the synthesis processes were performed in a glovebox with a dry inert Ar gas (the dew point < −100 °C, the oxygen concentration < 1 ppm).

### Crystal Structure Analysis

Crystalline phases were determined by XRD with the Bragg−Brentano geometry with a Cu Kα radiation source at RT. The lattice parameters were determined by the Pawley method using the TOPAS ver. 4.2 program (Karlsruhe, Germany: Bruker AXS GmbH). Rietveld analysis, where the fundamental parameter (FP) method was employed, was performed for crystal structure refinement. The microstructure of the bulks was evaluated using a field‐emission scanning electron microscopy (FE‐SEM; JSM‐7600F, JEOL) equipped with an energy‐dispersive spectrometer (EDS). The electronic structures were characterized by X‐ray photoemission spectroscopy (XPS) performed at the undulator beamline BL‐2A of the Photon Factory, High Energy Accelerators Research Organization (KEK). The binding energy was calibrated with the Fermi level of an evaporated reference Au film. Diffuse reflectance (*R*) spectra were measured at RT with a spectrophotometer in the wavelength (*λ*) range of 200−2400 nm. The obtained *R* spectra were converted using the Kubelka−Munk function (1−*R*)^2^/(2*R*) = *α*/*S*_f_, where *α* and *S*_f_ denote the optical absorption coefficient and the scattering factor, respectively, to obtain the quasi‐optical absorption spectra.

### Electronic and Thermal Properties

*σ* and *S* were simultaneously measured by the four‐probe method (ZEM‐3, ADVANCE RIKO, Inc.) under a He atmosphere. The *κ* was obtained from *κ* = *D*·*C*·*ρ*, where the thermal diffusivity (*D*) along the out‐of‐plane direction in the bulk was measured in an Ar atmosphere by a laser flash diffusivity method (LFA 457, NETZSCH) and the heat capacity (*C*) was measured by differential scanning calorimetry (DSCvesta, Rigaku Corp.), and the sample density (*ρ*) was determined by the dimensions and mass of the samples. The sound velocity (*v*_s_) is obtained by $v_{s}={\left(\frac{1}{3}\left[\frac{2}{v_{t}^{3}}+\frac{1}{v_{l}^{3}}\right]\right)}^{-1/3}$, where *v*_t_ and *v*_l_ are the transverse and longitudinal sound velocities measured by ultrasonic pulse‐echo method (1077DATA, KARL DEUTSCH) at RT. A detail of the phonon gas model analysis is described in the caption of Table S1 (Supporting Information).

### Density Functional Theory Calculation

The electronic structure calculations were performed for Ba_3_ *B*O models by DFT conducted using the projector augmented wave (PAW) method as implemented in the VASP code.^[^ ^34^, ^35^ ^]^ Ba [5*d*6*s*6*p*], Si [3*s*3*p*], Ge [4*s*4*p*], and O [2*s*2*p*] orbitals were included as valence states. The variable‐cell structure relaxations were performed by the generalized gradient approximation (GGA) Perdew–Burke–Ernzerhof (PBE) functional^[^ ^138^ ^]^ with a plane wave cut‐off energy of 550 eV, a Γ‐centered *k*‐mesh with the *k*‐spacing of 0.2 Å^−1^, as well as the convergence criteria of 10^−6^ eV for the energy and 0.01 eV Å^−1^ for the force. The relaxed lattice parameters are *a* = 7.762 Å, *b* = 10.844 Å, *c* = 7.569 Å for Ba_3_SiO and *a* = 7.761 Å, *b* = 10.888 Å, *c* = 7.608 Å for Ba_3_GeO, in consistence with the experimentally obtained values within 3% differences. The electronic band structures and DOSs were obtained by the HSE hybrid functional.^[^ ^36^ ^]^ The carrier effective masses were calculated by BoltzTraP2 code.^[^ ^139^ ^]^ The carrier transport properties of Ba_3_ *B*O were calculated by using DFT and DFPT as implemented in the Quantum ESPRESSO package.^[^ ^38^, ^39^ ^]^ The GBRV ultrasoft pseudopotentials^[^ ^140^ ^]^ were employed with the kinetic energy cutoff of 40 Ry (320 Ry) for wavefunctions (charge density). The *k*‐mesh density of 6×4×6 was used for the DFT calculations, and the 2×2×2 *q* points were used for the phonon and electron‐phonon calculations within DFPT. To compute the transport coefficients using dense *k* and *q* grids, the maximally localized Wannier functions (MLWFs) were constructed from the isolated 12 Kohn–Sham states below the VBM. The Wannierization was performed using the Wannier90 code,^[^ ^141^ ^]^ where the Si (Ge) *p* orbitals were used as initial projections and the outer energy window of [−2.0, 0] eV relative to the VBM. The *n*, *S*, *κ*_ele_, *σ*, and *τ*_e_ values at *T* = 300 K and 600 K were calculated using the PERTURBO code.^[^ ^41^ ^]^ The electron–phonon coupling coefficients were interpolated to the dense 120×80×120 *k* and *q* points and then used to solve the Boltzmann transport equation within the relaxation time approximation (RTA). The carrier lifetimes were computed from the imaginary part of the Fan–Migdal self‐energy, where the summation over the *q* points was performed by randomly sampling 10^6^ *q* points from a uniform distribution. It was confirmed that the transport coefficients reached converged with the above parameters.

The phonon transport calculations for Ba_3_ *B*O were performed using the ALAMODE code.^[^ ^51^, ^52^ ^]^ A 2×2×2 supercell (160 atoms) was used for the calculation of harmonic interatomic force constants (IFCs) and the anharmonic IFCs. The harmonic IFCs were determined by the finite‐displacement approach^[^ ^142^, ^143^ ^]^ and the anharmonic IFCs up to sixth‐order were estimated by the compressive sensing lattice dynamics. The temperature‐induced renormalized harmonic IFCs at *T* = 300 K were computed using the self‐consistent phonon (SCPH) theory,^[^ ^52^ ^]^ and were employed in the subsequent phonon transport calculations. All allowed interactions were included for the harmonic IFCs, the third‐order IFCs inside the cutoff radii of 12 bohr, and fourth‐, fifth‐, and sixth‐order IFCs inside the cutoff radii of 8 bohr. The DFT calculations to obtain the force were performed using the GGA‐PBE functional with a plane‐wave energy cutoff of 400 eV, a Γ‐centered 2×2×1 *k*‐mesh and an energy convergence criterion of 10^−8^ eV. *κ*_lat_ was calculated by solving the Peierls–Boltzmann transport equation under the RTA with a 7×7×5 *q* point mesh, which provides sufficient accuracy confirmed by the convergence tests (Figure S6, Supporting Information). The non‐analytic correction was included to the dynamical matrix by the mixed‐space approach,^[^ ^144^ ^]^ in which the Born effective charges of constituent elements and the dielectric constants were obtained by DFPT.^[^ ^37^ ^]^

CCDC 2291770 and 2291771 contain the supplementary crystallographic data for this paper. These data can be obtained free of charge from The Cambridge Crystallographic Data Centre via www.ccdc.cam.ac.uk/data_request/cif.

## Conflict of Interest

The authors declare no conflict of interest.

## Supporting information

Supporting Information

## Data Availability

The data that support the findings of this study are available from the corresponding author upon reasonable request.
