# Supplementary material for: Inverse‐Perovskite Ba3 BO (B = Si and Ge) as a High Performance Environmentally Benign Thermoelectric Material with Low Lattice Thermal Conductivity
Source: Adv Sci (Weinh). 2023 Dec 25;11(10):2307058. doi: 10.1002/advs.202307058 (PMC10933667; doi:10.1002/advs.202307058)
Supplement: Supplementary file 1 — Supporting Information [file ADVS-11-2307058-s001.pdf]

ADVANCED SCIENCE
Open Access

Supporting Information

for *Adv. Sci.*, DOI 10.1002/advs.202307058

Inverse-Perovskite Ba_3BO (B = Si and Ge) as a High Performance Environmentally Benign Thermoelectric Material with Low Lattice Thermal Conductivity

Xinyi He, Shigeru Kimura, Takayoshi Katase, Terumasa Tadano, Satoru Matsuishi, Makoto Minohara, Hidenori Hiramatsu, Hiroshi Kumigashira, Hideo Hosono and Toshio Kamiya**

© Copyright 2020. WILEY-VCH GmbH.

Supporting Information

Inverse-perovskite $Ba_3\mathit{B}O$ (B = Si and Ge) as a high performance environmentally benign thermoelectric material with low lattice thermal conductivity

Xinyi He, Shigeru Kimura, Takayoshi Katase, Terumasa Tadano, Satoru Matsuishi, Makoto Minohara, Hidenori Hiramatsu, Hiroshi Kumigashira, Hideo Hosono, and Toshio Kamiya**

Section 1. Crystal structure and microstructure analysis
Section 2. Carrier transport analysis
Section 3. Phonon transport analysis
Section 4. Convergence test for lattice thermal conductivity calculations

Section 1. Crystal structure and microstructure analysis

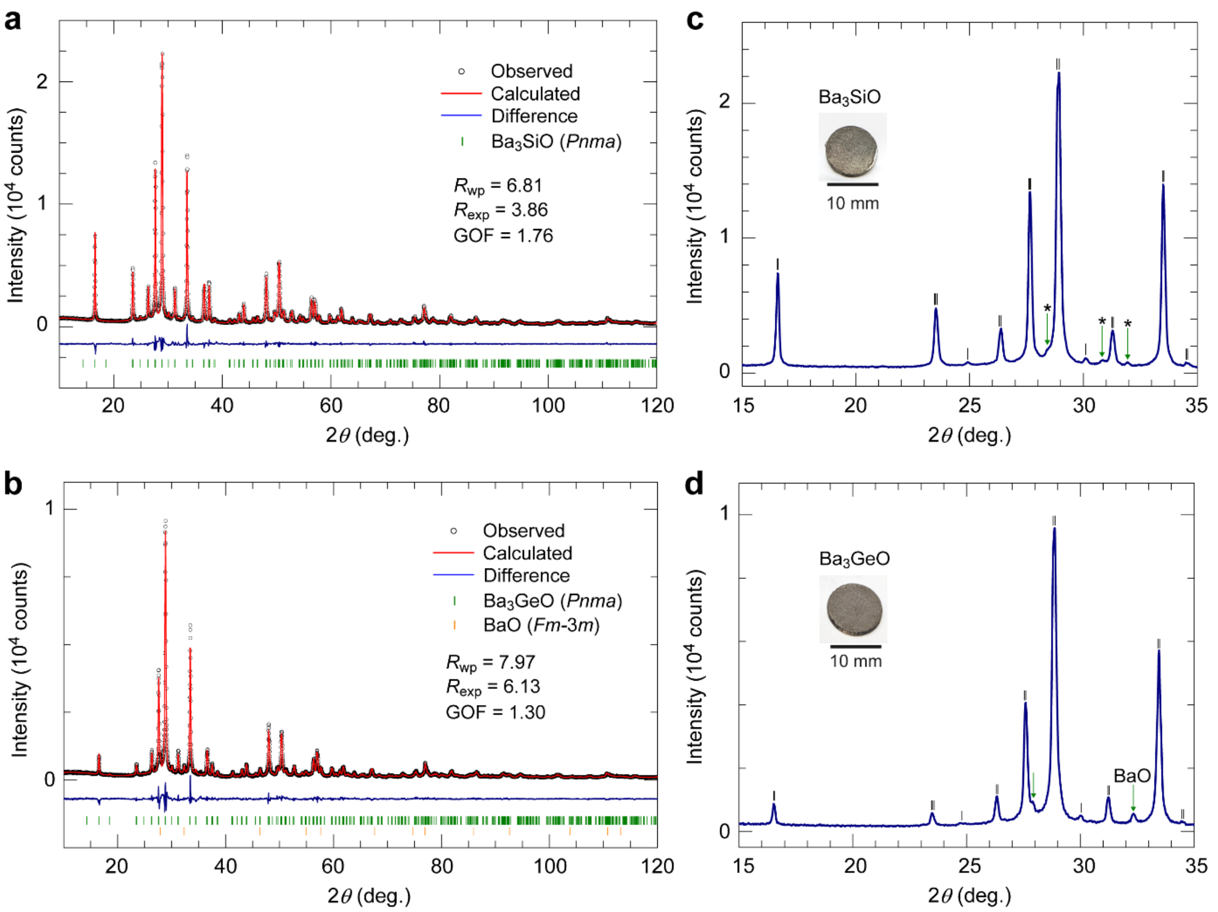

Figure S1. a,b) Rietveld analysis of XRD patterns for a) Ba_3SiO and b) Ba_3GeO bulk polycrystals. The green vertical bars indicate the diffraction angles from Ba_3SiO and Ba_3GeO phases (space group: *Pnma*). The orange vertical bars in (b) indicate the diffraction angles of BaO impurity. The crystallographic data after the Rietveld refinement can be obtained from CCDC 2291770 and 2291771. c,d) Expanded XRD patterns to show the existence of impurities. The black vertical bars denote the diffraction angles from the Ba_3SiO and Ba_3GeO phases. The diffraction peaks of BaO impurity are observed for the Ba_3GeO bulk, and the weak unidentified diffraction peaks (asterisk) are observed for the Ba_3SiO bulk. The photographs of Ba_3SiO and Ba_3GeO bulks are shown in the inset.

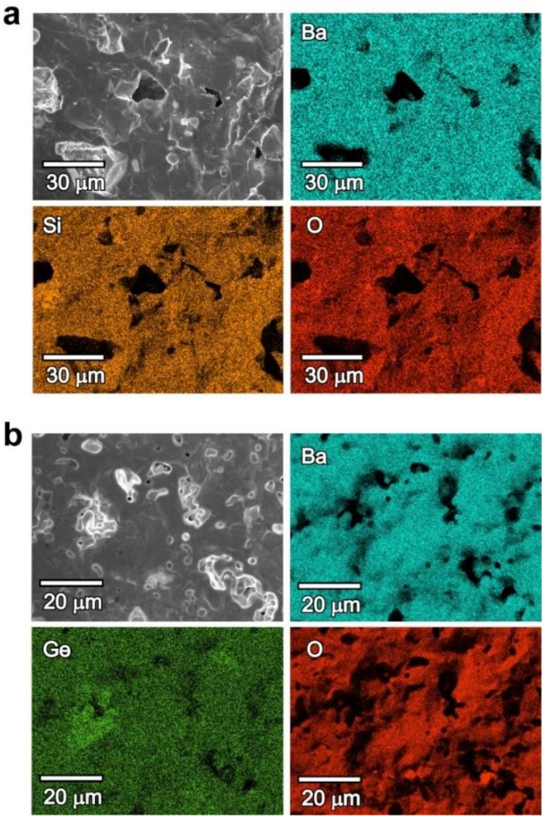

Figure S2. a,b) FE-SEM image and EDS mappings of Ba, Si, Ge, and O elements for polished surface of a) Ba_3SiO and b) Ba_3GeO bulk polycrystals. The sintered densities are 87% and 80% for Ba_3SiO and Ba_3GeO bulks, respectively. For Ba_3GeO, a slightly bright area was observed in the EDS map of Ge, suggesting the segregation of small amount of Ge impurity.

Section 2. Electronic structure and carrier transport analysis

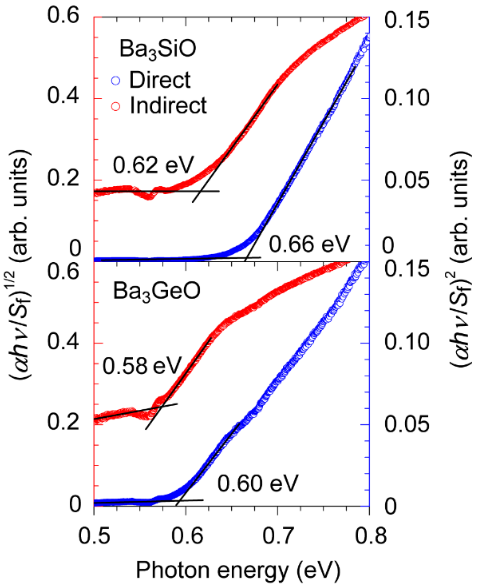

Figure S3. Kubelka-Munk plots of $(\alpha h\nu/S_f)^2$ for direct gap and $(\alpha h\nu/S_f)^{1/2}$ for indirect gap of Ba_3SiO (top panel) and Ba_3GeO bulk (bottom panel). α, S_f, h, and ν denote the optical absorption coefficient, scattering factor, Planck constant, and frequency, respectively. The direct E_g (indirect E_g) are 0.66 eV (0.62 eV) for Ba_3SiO, and 0.60 eV (0.58 eV) for Ba_3GeO, respectively, where the difference between the direct and the indirect E_g is small for both samples.

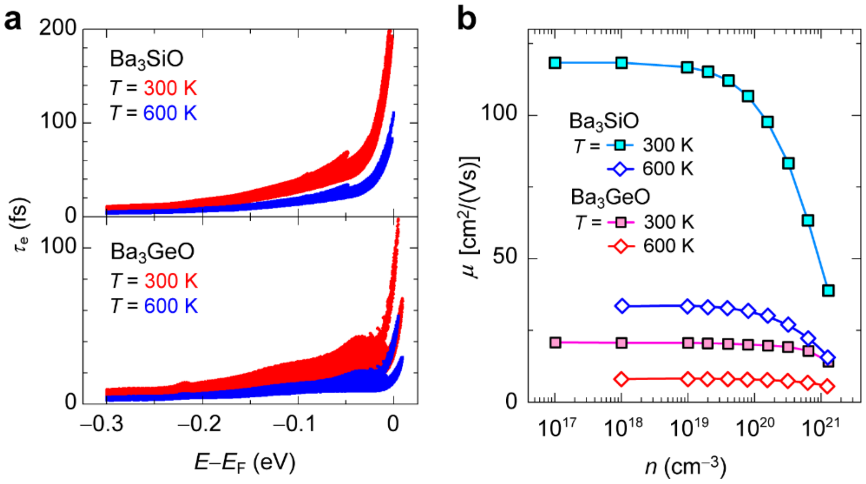

Figure S4. a) Calculated carrier lifetime (τ_e) as a function of energy from Fermi level (E–E_F) for Ba_3SiO (top panel) and Ba_3GeO (bottom panel) at T = 300 K and 600 K. b) Calculated carrier mobility as a function of carrier concentration (n) at T = 300 K and 600 K.

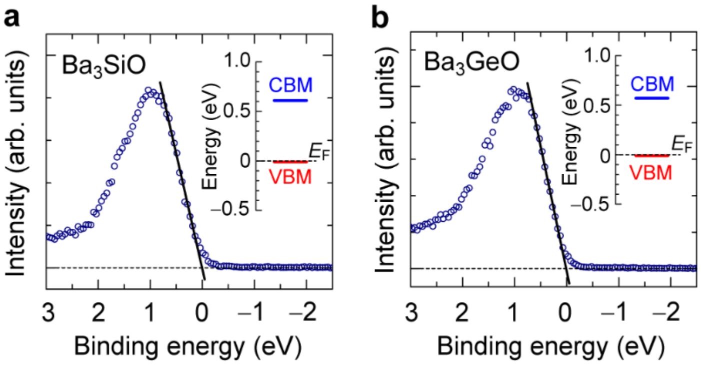

Figure S5. XPS spectra near valence band maximum (VBM) for a) Ba_3SiO and b) Ba_3GeO bulk polycrystals. The energy of VBM was estimated from the intersection between the extrapolated slope of the leading edge (solid line) and the background (dashed line). The inset shows the band alignment, where the conduction band minimum (CBM) and VBM are indicated by blue and red lines.

Section 3. Phonon transport analysis

Table S1. Elastic properties and phonon gas model analysis of Ba_3BO bulks. Those of $SrTiO_3$ bulk are also shown for comparison. v_t and v_l are the transverse and longitudinal sound velocities, measured by ultrasonic pulse-echo method. v_s is sound velocity obtained by $v_s = \left(\frac{1}{3}\left[\frac{2}{v_t^3}+\frac{1}{v_l^3}\right]\right)^{-1/3}$. G is the share modulus obtained by $G = \rho v_t^2$, and B is the bulk modulus obtained by $B = \rho\left(v_l^2 - \frac{4v_t^2}{3}\right)$, where the ρ is the sample density.[1-3] γ is the Grüneisen parameter obtained by $\gamma = \frac{3}{2}\left(\frac{1+r}{2-3r}\right)$, where r is the Poisson ratio expressed as $r = \frac{1-2(\frac{v_t}{v_l})^2}{2-2\left(\frac{v_t}{v_l}\right)^2}$.[4,5] Θ_D is Debye temperature obtained by $\Theta_D = \frac{h}{k_B}\left[\frac{3N}{4\pi V}\right]^{1/3} v_s$, where h is Planck's constant, k_B is the Boltzmann constant, N is the number of atoms in a unit cell, and V is the unit-cell volume.

	v_t (m/s)	v_l (m/s)	v_s (m/s)	G (GPa)	B (GPa)	γ	Θ_D (K)
Ba_3SiO	2095	3480	2317	59.8	92.8	1.348	219.6
Ba_3GeO	1787	3033	1981	51.8	80.0	1.426	187.4
$SrTiO_3$	4734	7951	5241	114.6	170.5	1.389	680.4

	κ_{lat} [W/(mK)]	C_V [MJ/(m^{3}K)]	τ_{ph} (ps)	l_{ph} (nm)
Ba_3SiO	1.00	3.41	0.16	0.38
Ba_3GeO	0.78	3.73	0.16	0.32
$SrTiO_3$	8.40	2.83	0.32	1.70

Section 4. Convergence test for lattice thermal conductivity calculations

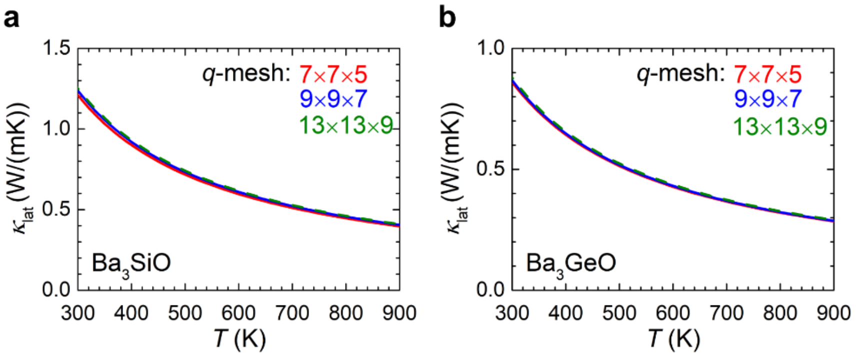

Figure S6. Convergence test in terms of the *q*-meshes for the lattice thermal conductivity (κ_{lat}) of a) Ba_3SiO and b) Ba_3GeO (averaged along *a*,*b*,*c*-axes).

References

[1] O. L. Anderson, *J. Phys. Chem. Solids* **1963**, *24*, 909−917.

[2] T. Górecki, *Mater. Sci. Eng.* **1980**, *43*, 225−230.

[3] M. Manikandan, A. Amudhavalli, R. Rajeswarapalanichamy, K. Iyakutti, *Solid State Commun.* **2019**, *291*, 36–42.

[4] P. H. Mott, J. R. Dorgan, C. M. Roland, *J. Sound Vib.* **2008**, *312*, 572−575.

[5] D. S. Sanditov, V. N. Belomestnykh, *Tech. Phys.* **2011**, *56*, 1619−1623.
